# First‐ and second‐generation biochemicals from sugars: biosynthesis of itaconic acid

**DOI:** 10.1111/1751-7915.12333

**Published:** 2015-12-07

**Authors:** Juan L Ramos, Zulema Udaondo, Baldomero Fernández, Carlos Molina, Abdelali Daddaoua, Ana Segura, Estrella Duque

**Affiliations:** ^1^ Biotechnology Technological AreaAbengoa ResearchC/Energía Solar s/n. Building E41014SevillaSpain

## Abstract

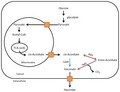

Competitive prices for sugars are needed to expand the range of biochemicals that can be industrially synthesized profitably. The most commonly accepted path from these starting materials to products is the Task 42 IEA BioEnergy biorefinery classification system, which is schematically depicted in Fig. 1. The upper part of the figure shows a wide range of potential raw material sources, while the lower part shows progressive series of chemicals that can be produced from C6 or C5 sugar core backbones through chemical catalysis or microbial fermentation. Subsequent modification of these compounds through chemical catalysis gives rise to yet another group of chemicals.

**Figure 1:**
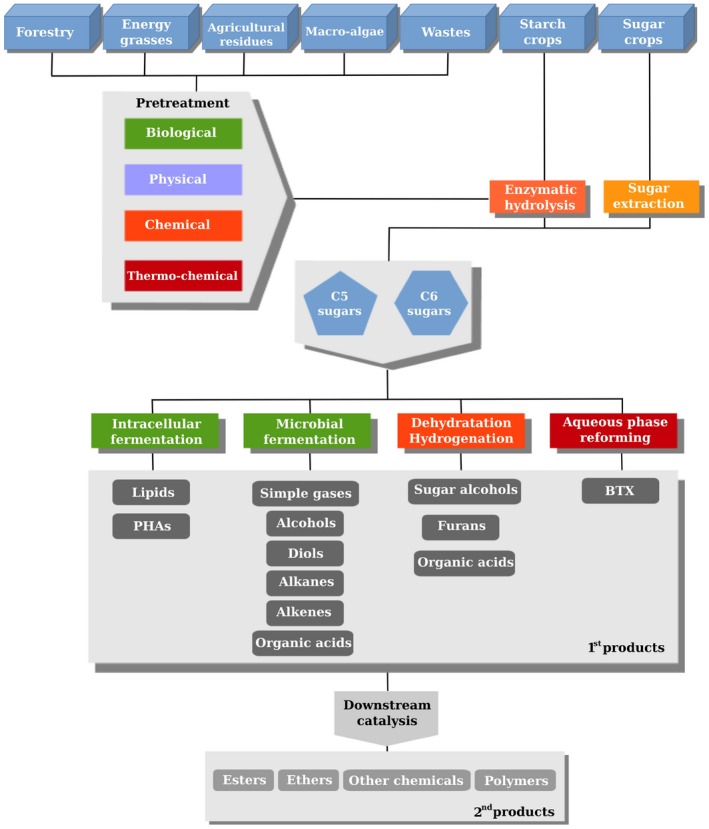
High‐level representation of pathways via the sugar platform. The aim of IEA (International Energy Agency) Bioenergy Task 42 is to initiate and actively promote information exchange on all features of biorefinery.

Sugars, which serve as the core structure upon which these chemicals are built, are typically generated using starch that originates from different grains. Of the total corn produced in the world, less than 5% is used for ethanol production. Given global controversy regarding the use of food for fuel production, many biofuel companies have begun to explore the production of ethanol from lignocellulosic materials, such as straw and other agricultural waste materials. Ethanol generated from starch is known as 1G ethanol, while ethanol produced from lignocellulose is known as 2G ethanol. We have adopted the terms 1G and 2G for use with biochemicals that can be made from starch and lignocellulose, respectively.

The use of lignocellulosic materials to produce biochemicals is challenging because these starting materials require intensive pretreatment (physical, chemical or biological) followed by enzymatic hydrolysis. The hydrolysis process is mediated by a set of enzymes, generically known as cellulases, which work synergistically to produce sugars. While glucose is almost the only product that results from starch hydrolyses, lignocellulose yields glucose in addition to a range of other sugars, such as xylose, arabinose, rhamnose and galactose. Regardless of whether the sugars are derived from starch or lignocellulose, fermentation of the sugars can produce downstream products than include alcohols, organic acids, alkenes, lipids and a wide range of other chemicals. This conversion can be accomplished using bacteria, fungi or yeast (genetically modified or not) using a variety of process conditions (e.g. low/high pH, aerobic/anaerobic, mesophilic/thermophilic and various nutritional regimes). The biotransformation industry modifies these and other variables to develop new processes through iterative parameter optimization with one aim: to attain the highest possible biochemical yields at rates that enable maximal recovery after downstream processing.

The number of biochemicals currently produced through this manner at a commercial scale is still low. Examples of these include: ethanol, lactic acid, succinic acid, butanol, acetone, sorbitol and itaconic acid. In this issue of *Microbial Biotechnology*, a new pathway for production of itaconic acid is described by Geiser *et al*. (2015). The annual production of itaconic acid, an unsaturated dicarboxylic acid, is around 50 000 tons/year. This biochemical is used as building block for the biosynthesis of pharmaceuticals, certain resins and adhesives (Steiger *et al*., 2013). It should be noted that poly (itaconic acid) can be used to develop superabsorbents, anti‐scaling agents in water treatment, and can comprise components of detergents and dispersants (Klement and Büchs, 2013). Because of its industrial potential, itaconic acid was selected by US Department of Energy as one of the top 12 chemical candidates (that can be generated from biomass) to serve as a building block for the production of value added chemicals (Werpy and Petersen, 2004). The existing itaconic biosynthesis pathway was first studied in *Aspergillus terreus*. Early studies established that itaconic acid was produced from *cis*‐aconitate, a tricarboxylic acid cycle intermediate, via the action of a decarboxylase known as CadA (Okabe *et al*., 2009; Klement and Büchs, 2013) (Fig. 2). The microbial‐based production of itaconic acid has been reported to be as high as 45–80 g l^−1^ of media (Kanamasa *et al*., 2008; Steiger *et al*., 2013). In addition to *Aspergillus*, previous studies have shown that other fungi, such as *Ustilago maydis*, produce itaconic acid.

**Figure 2:**
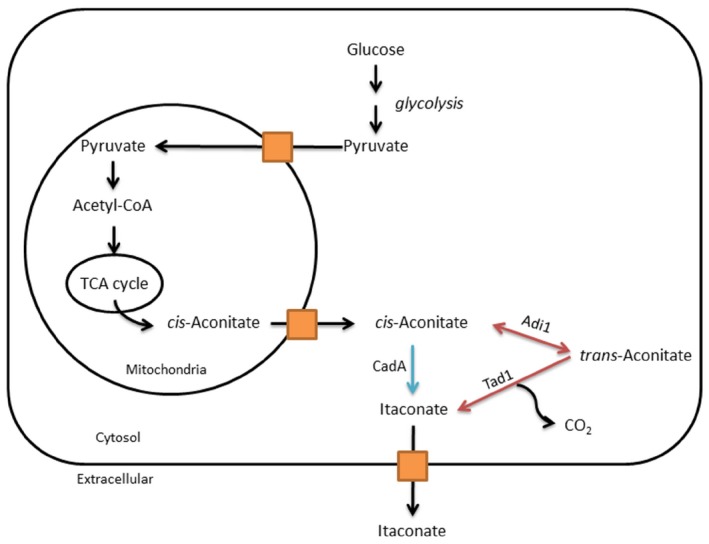
Pathways for itaconic acid biosynthesis. *cis*‐Aconitate is an output chemical from the tricarboxylic acid (TCA) cycle. Blue lines represent the classic pathway described in *A* *spergillus* species (Huang *et al.,* 2014). Red lines represent the new pathway described in *U* *stilago maydis* by Geiser *et al*. (2015).

The new pathway revealed by Geiser *et al*. (2015) has been identified in *U. maydis* and it involves the isomerisation of *cis*‐ to *trans*‐aconitate, via a cytosolic aconitase isomerase (Adi1), followed by a decarboxylation step mediated by a novel decarboxylase (Tad1), which exhibits significant sequence similarities to lactonizing enzymes. A quick BLASTp search reveals that the isomerase described in this study and the new decarboxylase are present in a limited number of fungi with the best hit with sequences from *Pseudozyma hubeiensis*. The production rates reported for *U. maydis* are lower than those from *A. terreus*; however, gene regulation studies and optimization of production conditions in *U. maydis* are needed to reveal the biotechnological potential of the new pathway.

The current limitation of the biological production of itaconic acid on an industrial scale seems to be production costs (Klement and Büchs, 2013). This limitation can be overcome through improving microbial strains, optimization of processes and the sourcing of cheaper raw materials. The newly identified pathway also provides new options for optimizing production processes, and moves us one step closer to overcoming the current affordability challenges.

## Conflict of Interest

None declared.
